# Misdiagnosed tuberculosis being corrected as *Nocardia farcinica* infection by metagenomic sequencing: a case report

**DOI:** 10.1186/s12879-021-06436-6

**Published:** 2021-08-04

**Authors:** Lei Pan, Xiao-hong Pan, Jie-kun Xu, Xiao-qing Huang, Jun-ke Qiu, Cai-hong Wang, Xiao-bo Ji, Yang Zhou, Min-jie Mao

**Affiliations:** 1 Department of Tuberculosis Intensive Care Unit, Affiliated Hangzhou Chest Hospital, Zhejiang University School of Medicine, No. 208 Huancheng Dong Road, Hangzhou, 310003 China; 2 BGI PathoGenesis Pharmaceutical Technology, BGI-Shenzhen, Shenzhen, 518083 China

**Keywords:** *Nocardia farcinica*, Tuberculosis, Metagenomics, Misdiagnosis

## Abstract

**Background:**

Disseminated nocardiosis is liable to be misdiagnosed owing to the non-specific clinical manifestations and laboratory/imaging findings. Metagenomic next-generation sequencing (mNGS) is a culture-independent and rapid method for direct identification of all microorganisms in clinical specimens.

**Case presentation:**

A 72-year-old man was admitted to our hospital on February 20, 2019 with a history of recurrent cough, expectoration, fever, and diarrhea since 1 month, and unconsciousness since 1 week. Contrast-enhanced magnetic resonance imaging of head showed multiple lesions in the bilateral cerebral hemispheres, brainstem, and cerebellar hemispheres. The presumptive diagnosis was disseminated tuberculosis, although all tests for mycobacterium were negative. However, the patient did not benefit from antituberculosis treatment. Repeat MRI showed multiple abnormal signals in the brain and progression of meningeal thickening. Cerebrospinal fluid and bronchoalveolar lavage fluid specimens were subsequently sent for PMSeq metagenomics sequencing; the results indicated *Nocardia. farcinica* as the predominant pathogen. The anti-TB treatment was stopped and the patient was prescribed sulphamethoxazole in combination with linezolid and meropenem for nocardiosis. He showed gradual neurological improvement and was transferred to Huashan Hospital. He was discharged from the hospital on April 19, 2019, but died of persistent diarrhea on May 26, 2019.

**Conclusions:**

Patients with suspected nocardiosis do not always respond to conventional treatment; therefore, mNGS can facilitate diagnosis and timely treatment decision-making.

## Background

The genus *Nocardia* includes at least 50 species, approximately half of which are pathogenic to humans or animals [1]. This bacterium invades the human body mainly through the respiratory tract, and occasionally through the skin and digestive tract [2]. Approximately 50% of cases of nocardiosis occur in immunocompromised settings such as in patients with tumors, chronic obstructive pulmonary disease, AIDS, organ transplant recipients, and those on long-term steroid or immunosuppressant therapies [3, 4]. *Nocardia asteroid* is the most prevalent species which accounts for more than 70% of infections, while *Nocardia farcinica* is more likely to invade immunocompetent hosts than the other *Nocardia* species [5–9]. It mainly causes lung and systemic infection [10–12], but may even cause brain abscess [5]. Kumar et al. reviewed the case reports of nocardiosis published from 1966 to 2011; of these, 41 cases of brain abscess were caused by *Nocardia farcinica* [10–14]*.* In the absence of timely treatment, *Nocardia farcinica* infection is usually life-threatening. The general mortality rate is 14 to 40%, while the mortality rate associated with disseminated infection may be as high as 100% [15].

Owing to the non-specific clinical manifestations, *Nocardia* infection is liable to be misdiagnosed as tuberculosis, fungal pulmonary disease, or lung malignancy [16]. The diagnosis of *Nocardia* infection is based on pathogen culture and biochemical identification [17]. However, it normally takes at least 2–7 days of 37 °C aerobic culture for *Nocardia* to exhibit slow growth and up to 4–6 weeks to develop into visible colonies. Biochemical identification may take even longer. Therefore, it is typically difficult to isolate and identify *Nocardia*, especially if the specimen is contaminated with other microorganisms.

Metagenomic next-generation sequencing (mNGS) is a culture-independent method that allows identification of all microorganisms in environmental or clinical samples by high-throughput sequencing and genomics analysis of total DNA [18–20]. In addition, mNGS takes only approximately 24 h from sampling, nucleic acid extraction, library sequencing, data processing and reporting compared to conventional methods; therefore, it allows for quick identification of pathogens in case of unexplained diseases. We utilized this platform to successfully identify *Nocardia* farcinica as the main cause of severe disseminated infection in a elderly man, who was initially misdiagnosed as a case of tuberculosis.

## Case presentation

**Fig. 1:**
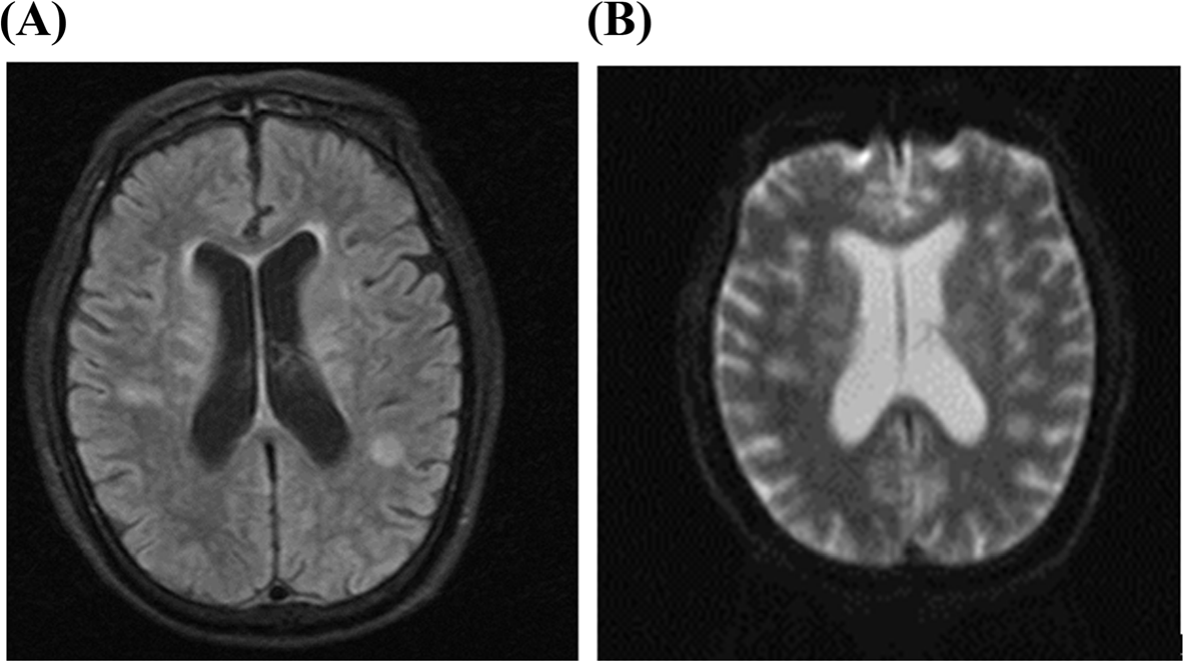
(**A** and **B**) Contrast-enhanced magnetic resonance images of head obtained at admission (February 20, 2019). Multiple bilateral lesions are seen in the cerebral hemispheres, brainstem, and cerebellar hemispheres

**Fig. 2:**
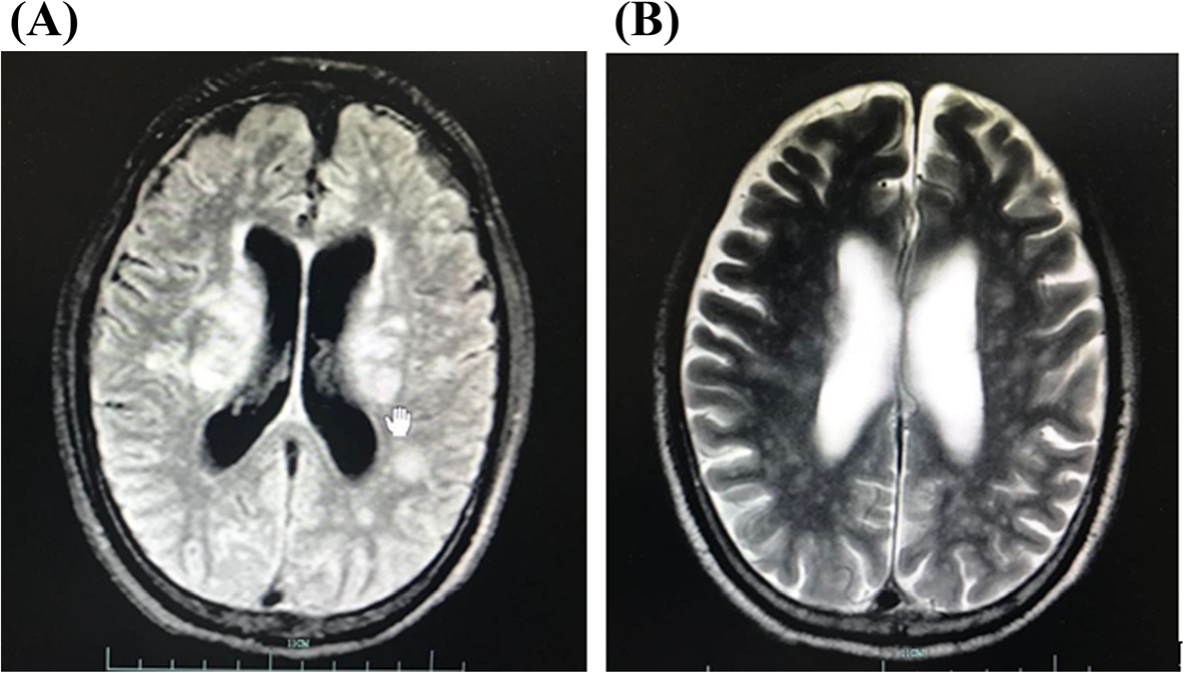
(**A** and **B**) Repeat head MRI (dated: February 24, 2019) showing multiple abnormal signals in the brain and progression of meningeal thickening

A 72-year-old male developed cough, sputum, coryza, fever with chills (body temperature: 37–38 °C), diarrhea, fatigue, and poor appetite since January 2019. However, the patient did not actively seek treatment for these symptoms. On February 13, 2019, the patient experienced an episode of fainting in the toilet followed by spontaneous recovery and he was admitted to the Yuhang District Traditional Chinese Medicine Hospital. Results of laboratory investigations were as follows: hemoglobin, 60 g/L; serum potassium, 2.32 mmol/L; serum C-reactive protein (CRP), 74 mg/L; serum albumin, 28.3 g/L; serum creatinine, 179.8 μmol/L. On the following night, the patient suddenly became unconscious and was referred to the emergency department of the Second Affiliated Hospital of Zhejiang University. The patient was administered empirical and supportive treatment, including antibiotics (sulperazon), respiratory decongestants, and blood transfusion. On February 20, 2019, the patient was admitted to our hospital with chief complaints of recurrent cough, expectoration, fever, and diarrhea for 1 month, and unconsciousness for 1 week. Throat swab culture and influenza virus nucleic acid screening tests were both negative. Contrast-enhanced magnetic resonance imaging of head showed multiple bilateral lesions in the cerebral hemispheres, brainstem, and cerebellar hemispheres (Fig. 1). Physical examination findings at admission were: body temperature, 38.3 °C; heart rate 103 beats/minute, respiratory rate: 18/min, BP 138/73 mmHg, SpO2 94%. The patient was comatose with Glasgow Coma Scale (GCS) score of 1 + 1 + 1. The main differential diagnosis at admission was disseminated tuberculosis, including tuberculous meningoencephalitis, lymph node tuberculosis, intestinal tuberculosis. The cerebrospinal fluid, bronchoalveolar lavage fluid (BALF), and sputum samples were examined on several occasions. Simultaneous amplification and testing for *Mycobacterium tuberculosis* (SAT_TB) test [21] and routine TaqMan Real-Time PCR assay were separately used to detect mycobacteria RNA and DNA in the above specimens. However, the results of acid-fast staining, tuberculosis RNA, mycobacterium DNA, and GeneXpert were negative. Bacterial, mycobacterial and fungal cultures of cerebrospinal fluid were also negative. After obtaining the consent of family members, the patient was administered a trial of antituberculosis treatment (isoniazid and rifampicin, 600 mg per day, respectively). The patient also received supportive treatment, including dexamethasone to alleviate the intracranial inflammatory response. However, there was no improvement in the consciousness level (GCS score: 1 + 1 + 1). On February 24, 2019, repeat head MRI showed multiple abnormal signals in the brain and progression of meningeal thickening (Fig. 2). In order to confirm the diagnosis, his cerebrospinal fluid specimen was sent to the Wuhan BGI clinical laboratory for PMSeq metagenomics sequencing (https://en-medical-PMSeqs.html) on February 24, 2019. The qualified libraries were constructed using improved DNA Nanoballs (DNB) technology and sequenced on the BGISEQ-50 platform.

Specifically, 0.5–3 mL CSF/BALF sample from the patient was collected according to standard procedures. 0.5 mL sample and 1 g 0.5 mm glass bead were mixed in a 1.5 mL microcentrifuge tube and attached to a horizontal platform on a vortex mixer. After vigorous agitation at 2800–3200 rpm for 30 min, 0.3 mL sample was transferred into a new 1.5 mL microcentrifuge tube and DNA was extracted using the TIANamp Micro DNA Kit (DP316, TIANGEN BIOTECH) according to the manufacturer’s protocol. DNA libraries were constructed through DNA-fragmentation, end-repair, adapter-ligation, and PCR amplification. Agilent 2100 and Qubit was used for quality control of the DNA libraries (200–300 bp, > 2 ng/μL). Quality qualified libraries were sequenced using the BGISEQ-50 platform. High-quality sequencing data were generated by removing low-quality reads, followed by computational subtraction of human host sequences mapped to the human reference genome (hg19) using Burrows-Wheeler Alignment. After removal of low-complexity reads, the remaining data were classified by simultaneously aligning to four Microbial Genome Databases, consisting of bacteria, fungi, viruses and parasites. The classification reference databases were downloaded from the NCBI (ftp://ftp.ncbi.nlm.nih.gov/genomes/). RefSeq contains 4945 whole genome sequences of viral taxa, 6350 bacterial genomes or scaffolds, 1064 fungi related to human infection, and 234 parasites associated with human diseases. The detection of pathogen was reported based on the following criteria: 1) the detected microorganisms were related to human infections, which were confirmed by published clinical reports and records; 2) the sequence number of the detected pathogens in the sample should exceed that in the blank control; 3) the sequence number of pathogenic bacteria (except MTB complex), fungi or viruses, should be > 3; 4) the sequence number of pathogenic parasites should be > 100; 5) MTB complex was considered positively detected with more than 1 sequence. Owing to the difficulty in DNA extraction from MTB complex, these pathogens have lower threshold than the other pathogens.

**Fig. 3:**
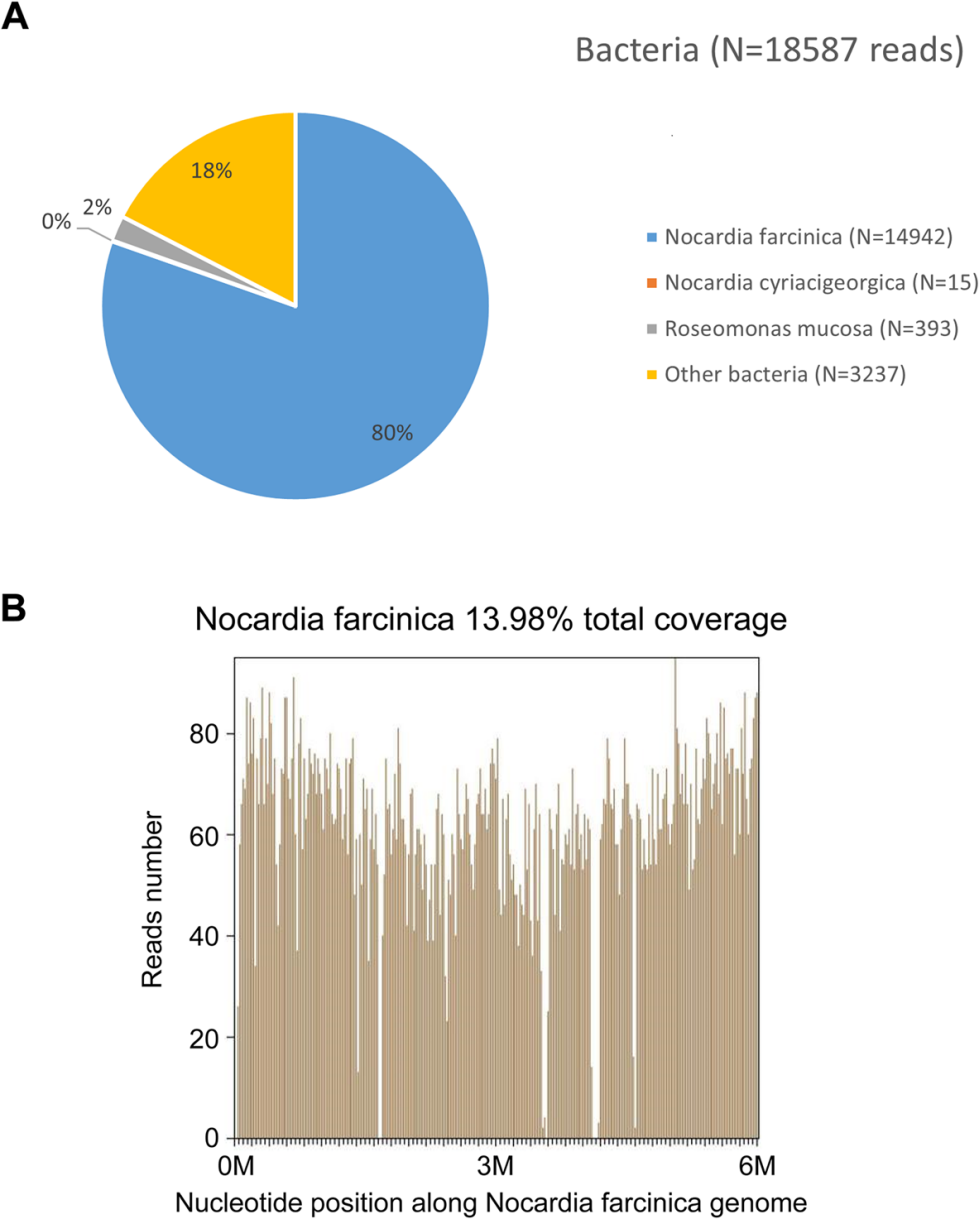
**(A)** Results of next-generation sequencing for pathogen detection in cerebrospinal fluid (dated: February 26, 2019): *Nocardia. farcinica* was identified as the predominant pathogen (6775 sequence reads); other pathogens identified were *Nocardia. cyriacigeorgica* (7 sequence reads) and *Mycobacterium tuberculosis* complex (1 sequence read). **(B)** The reads coverage of *Nocardia. farcinica* was 13.96%

**Fig. 4:**
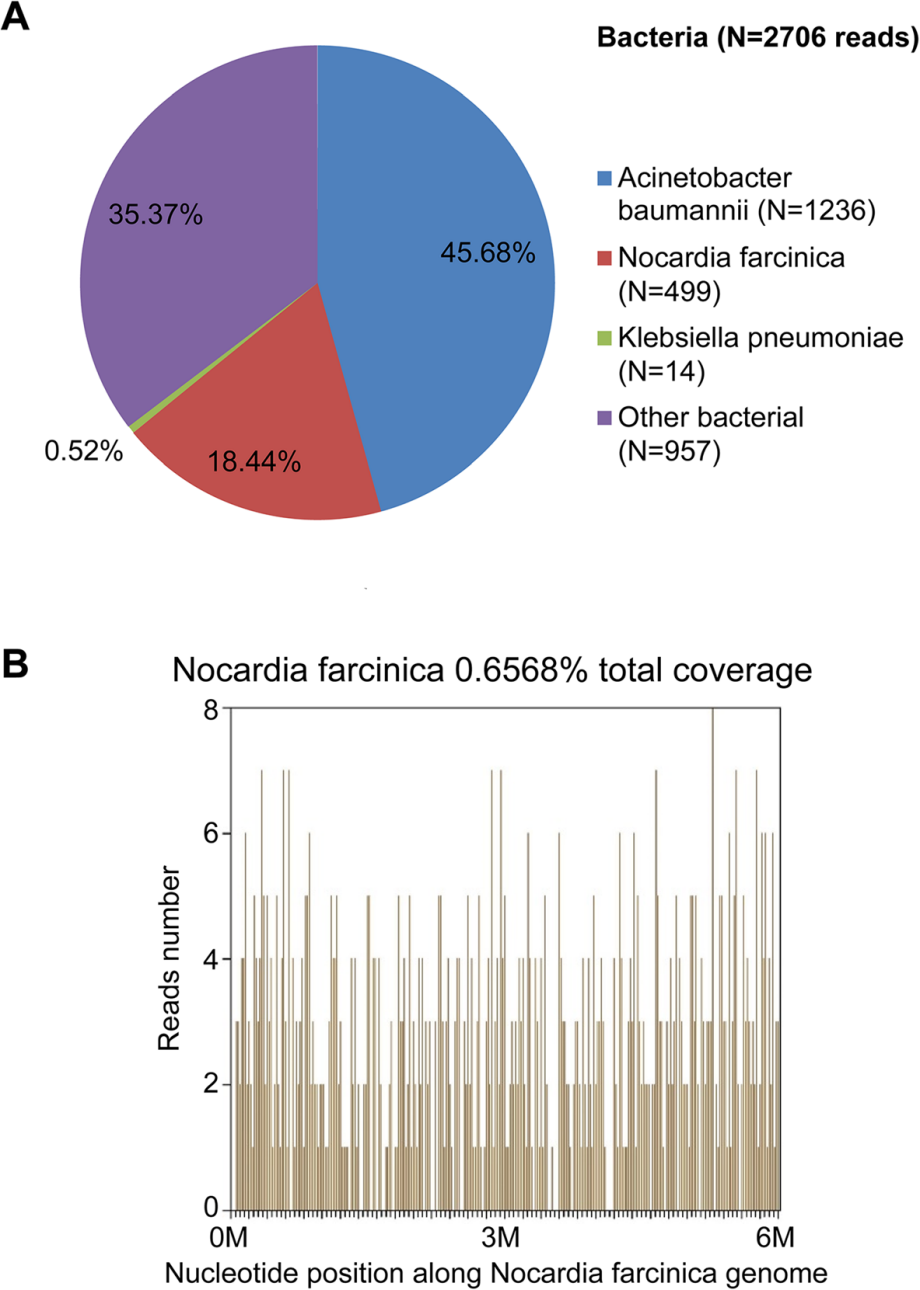
**(A)** Results of next-generation sequencing for pathogen detection in bronchoalveolar lavage fluid (dated: March 05, 2019) identified *Nocardia. farcinica* (499 sequence reads); *Mycobacterium tuberculosis* complex was not identified. **(B)** The reads coverage of *Nocardia farcinica* was 0.66%

**Fig. 5:**
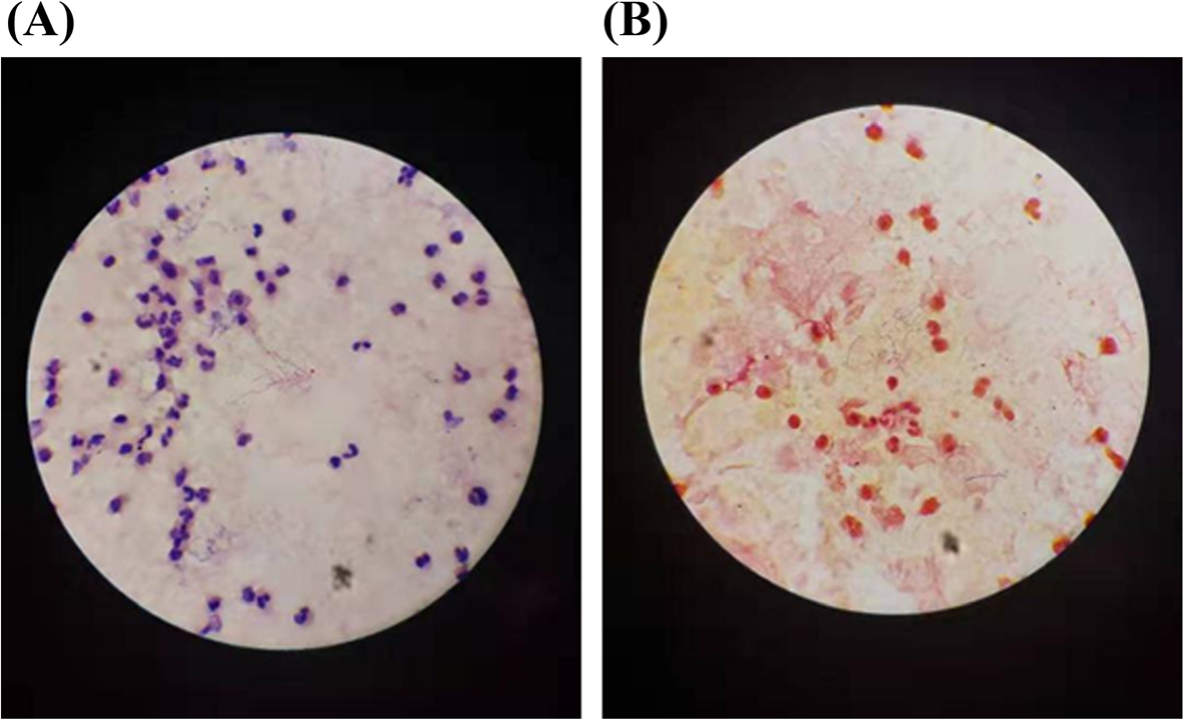
Smear staining of bronchoalveolar lavage fluid showing gram-variable branched bacilli **(A)** with weakly positive acid-fast staining **(B)**. The findings are consistent with the morphological features of nocardia

On February 26, 2019, NGS identified *Nocardia farcinica* as the predominant pathogen (6775 sequence reads); the other pathogens identified were *Nocardia. cyriacigeorgica* (7 sequence reads) (Fig. 3) and *Mycobacterium tuberculosis* complex (1 sequence read). The patient was then administered sulphamethoxazole (SMZ) in combination with linezolid and meropenem for anti-nocardiosis treatment; in addition, the anti-TB treatment was continued. Results of pathogen detection in BALF (March 05, 2019) identified *Nocardia. farcinica* (499 sequence reads); however, no *Mycobacterium tuberculosis* complex was identified (Fig. 4). These results suggested that the single sequence read of tuberculosis found in the cerebrospinal fluid was the result of contamination; therefore, the anti-TB treatment was discontinued. Smear staining of BALF showed gram-variable branched bacilli with weakly positive acid-fast staining (Fig. 5), which was consistent with the morphological features of *Nocardia*.

On March 11, 2019, the patient was transferred to the Huashan Hospital of Fudan University. At that time, the patient’s consciousness and tingling sensations had improved (GCS score: 2 + 1 + 3). The patient was prescribed meropenem (2 g, 8 hourly) in combination with SMZ (2 tablets, 8 hourly) for anti-nocardiosis treatment. He was also administered repeated infusions of red blood cell suspension, platelets, and injections of gamma globulin. The patient showed gradual improvement with recovery of consciousness (sensitive to painful stimuli) and was able to exercise. He was discharged from the hospital on April 19, 2019 and was prescribed 3 L/min oxygen inhalation with a metal cannula.

The patient was intermittently followed up telephonically and via the WeChat application. His family members reported that the patient developed persistent diarrhea on May 26, 2019, and was taken to a local hospital in an unconscious state. His blood pressure was not recordable and he died prior to any active treatment.

## Discussion and conclusions

Currently, less than 1% of bacteria can be identified by isolation and culture methods [22]. Microscopic observation allows for morphological identification; however, it has low sensitivity and specificity [23]. Serological diagnosis is prone to cross-reactivity and has poor specificity [24]. Polymerase chain reaction (PCR) method fails to detect unknown and highly variable pathogens [25]. Therefore, in up to 40% cases of gastroenteritis and 60% cases of encephalitis, the causative pathogen cannot be determined [26, 27]. In the 1990s, Handelsman et al. pioneered the concept of metagenome. Subsequently, metagenomics was defined as a research method that entails application of modern sequencing technology for direct study of the microbial community in their natural state without isolation and culture [18–20]. In recent years, metagenomics has played an increasingly important role in pathogen detection. In a retrospective study of an acute intestinal hemorrhagic epidemic outbreak in Germany, Loman et al. [28] collected 40 samples of Shiga toxin-producing *Escherichia coli* (STEC)-positive feces and 5 control fecal samples from patients with STEC-negative diarrhea. The pathogenic strain of the disease, STEC O104: H4, was finally detected in samples of STEC-positive patients by metagenomic sequencing without involving isolation and culture; in addition, the Shiga toxin-producing gene fragments were also detected. In 2014, Fisher et al. [29] collected BALF sample from a patient with acute respiratory distress syndrome (ARDS) in Germany. The extracted DNA was subjected to NGS and the causative pathogen was quickly identified as *Chlamydia psittaci* (within 50 h). Subsequently, the test results were verified using PCR and the patient recovered after administration of specific antibiotics. Various pathogenic microorganisms such as viruses, bacteria, and fungi can cause meningitis; however, the traditional methods for identification of causative pathogen are time-intensive. Guan et al. reported the application of metagenomics to facilitate the diagnosis of suspected viral meningitis. They detected herpes simplex virus type 1 (HSV-1) after NGS sequencing of cerebrospinal fluid samples of 2 patients. Herpes simplex virus type 2 (HSV-2) and human herpes virus type 3 (HHV-3) were detected in the samples of two other patients; all 3 patients were subsequently confirmed by PCR [30]. Yao et al. reported 3 cases of suspected *Listeria* meningitis in whom the culture results were negative. Sequencing study of the cerebrospinal fluid identified the *Listeria* sequence, which was further verified by PCR [31]. The above studies demonstrate that metagenomics can rapidly detect rare pathogen infections, which is essential for the diagnosis and treatment of infectious meningitis.

However, mNGS is not the gold standard but a supplementary method for pathogen screening. Although mNGS can detect more difficult-to-culture (DTC) bacterial pathogens in a single run than conventional methods, its diagnostic ability is still impacted by several factors including sample types, sample processing method, sequencing platforms, and strategies. Thus, it may have a lower detection sensitivity than PCR under some circumstances. In addition, mNGS detection is relatively costly, which may impose a financial burden on the patients and the public health system. Therefore, mNGS should only be used to identify potential pathogens when routine laboratory tests, imaging, and pathological assays cannot provide the basis for effective anti-infective treatment.

In our patient, the clinical manifestations and imaging findings strongly suggested an infection of the central nervous system; however, the results of routine tests for detection of pathogenic microorganism were all negative. In addition, patient did not benefit from anti-tuberculosis treatment, which prompted us to use the metagenomics platform for rapid identification of the causative pathogen. *Nocardia* does not normally colonize the human body and there is limited scope for laboratory contamination; therefore, detection of *Nocardia* in clinical samples should be considered indicative of infection [32]. The significant read number and genomic coverage of *Nocardia farcinica* indicated the diagnosis of nocardiosis. SMZ is still the first-choice drug for empirical treatment of nocardiosis; this is because of its good permeability in most tissues (including the central nervous system) and the high serum concentration achieved after oral administration. However, owing to the development of drug resistance, SMZ-based combination therapy is recommended for patients without pathogen identification and antibiotic sensitivity results, especially those with severe infection [15, 33, 34]. Our patient was prescribed SMZ in combination with linezolid and meropenem. The diagnosis of nocardiosis was also supported by the efficacy of anti-Nocardia treatment.

The reported mortality rate associated with nocardiosis is 14–40%; however, it can be as high as 40–87% in patients with hematogenous spread of infection to the brain [15, 33, 34]. The overall prognosis is related to the general condition of the patient, the timeliness of treatment, the spread of infection, and the degree of drug resistance. Our patient suffered from disseminated nocardiosis and showed resolution of neurological symptoms after correct treatment. However, he died of persistent diarrhea on May 26, 2019. It is not certain whether the persistent diarrhea was caused by Nocardiosis, but the poor outcome is consistent with the high mortality rate associated with Nocardiosis in immunodeficient patients [15].

There were two limitations in the present study. At first, due to the need for timeliness and cost-effectiveness of pathogen detection, the library was sequenced only once. In addition, pathogen-specific PCR tests and Sanger sequencing were not conducted to confirm the presence of the pathogen in the sample. We expect to refine these screen-confirmation processes in future investigation.

## Data Availability

The datasets generated and analyzed during the present study are available from the corresponding author on reasonable request.

## Abbreviations

mNGS: Metagenomic next-generation sequencing
MRI: Magnetic resonance imaging
BALF: Bronchoalveolar lavage fluid
CRP: C-reactive protein
GCS: Glasgow Coma Scale
SMZ: Sulphamethoxazole
PCR: Polymerase chain reaction
STEC: Shiga toxin-producing *Escherichia coli*
ARDS: Acute respiratory distress syndrome
HSV-1: Herpes simplex virus type 1
HSV-2: Herpes simplex virus type 2
HHV-3: Human herpes virus type 3
